# Representations of Low(er) Alcohol (Craft) Beer in the United States

**DOI:** 10.3390/nu14234952

**Published:** 2022-11-22

**Authors:** Colleen C. Myles, Bren Vander Weil, Delorean Wiley, Bart Watson

**Affiliations:** 1 Department of Geography and Environmental Studies, Texas State University, San Marcos, TX 78666, USA; 2 Brewers Association, Boulder, CO 80302, USA

**Keywords:** craft beer, low alcohol beer, non-alcoholic beer, social trends, qualitatively-informed quantitative analysis

## Abstract

Given increasing social interest in health and wellness, rising cultural trends toward sobriety and moderating alcohol intake, and improvements in brewing technology, low(er) alcohol beer is a rising segment in the beer industry for both craft and larger-scale producers. In this paper, we assess the representation of low(er) alcohol beer among craft brewers in the United States. Using a novel quantitatively-informed qualitative analytical approach, we surveyed a randomized, non-representative sample of 400 craft brewery websites in the United States to assess the relative presence of low(er) alcohol beers as well as how these brews are represented by the breweries themselves. To do so, we recorded, both numerically and via website screenshots, the lowest ABV beverage on offer and noted the beer type, the beer name, and the ABV. Ales were the most prominent style of beer on offer, accounting for 62% of the low(er) ABV beers identified. Only 15.5% of the breweries surveyed in this study offered a beer with an ABV of less than 4%; however, an additional 67.9% offered a beer with an ABV of less than 5%. The representations of these low(er) alcohol products focused mostly on taste, health, and demographic indicators.

## 1. Introduction

Due to increasing social interest in health and wellness, particularly vis-à-vis alcohol consumption, rising cultural trends toward sobriety and moderating alcohol intake, and improvements in brewing technology [1,2], “low-gravity” beer is on the rise among both craft and larger-scale producers [3]. As this particular style serves to distinguish breweries from their competition, in this paper, we assess the representation of “NoLo” [2] (no/low-alcohol) beer among craft brewers in the United States.

Cultural trends and available evidence indicate that there is a renaissance occurring among low- and no-alcohol beers in the United States [3,4,5]. While this is not the first time that these categories of beer have grown and gained share in the United States, the current market changes taking place now look different for a variety of reasons. Namely, the customers in this segment, and the market itself, are positioned differently than in the past. For example, even though non-alcoholic beers reached a 1.2% share of the US beer market in the early 1990s [6], the current movement (as of the end of 2022) currently accounts for just 0.64% of the US market share [7]; however, it is distinctive insofar as it is oriented toward a different kind of consumer. Historically, the customer base, and marketing aimed toward them, was often focused on non-drinkers, those who, for health other reasons, were avoiding alcohol altogether. However, in more recent years, there are many signs that the emergence of non-alcoholic products—not only beer but also wine and hard liquor—overlaps more with customers who also drink alcoholic beverages, such as full-strength beer. These are consumers who drink non-alcoholic beers on particular occasions when they want to abstain (times of day, days of the week) or use non-alcoholic products in combination with alcoholic products to moderate their overall consumption [8]. Moreover, increasingly, the no/low-alcohol market is driven by flavor rather than by “refreshment” cues that mirror those of the lager and light lager markets [9], creating opportunities for small (craft) producers to leverage taste (i.e., flavor or style variety) to enter the market [5,8], especially when these low(er) alcohol products can be used to appeal to customers focused on health and wellness and/or those seeking to reduce their overall alcohol intake [10,11].

Similarly, while low-alcohol beers were previously a fairly large market in the United States—as recently as 2009, they represented 2% of the US beer market [12]—those beers were a part of the market mainly due to the regulatory context in their place of production [13]. For example, certain states had “3.2 rules” (3.2% being the alcohol by weight [ABW], so up to 4% alcohol by volume [ABV]) that facilitated consumption by younger consumers (typically at age 18) or ensured wider accessibility (i.e., these products could be sold in more locations than higher alcohol products could be) [13,14]. While there is no formal definition for “low-alcohol” beer in the United States [2,15], a commonly used indicator mirrors these “3.2” laws and dates back to the repeal of Prohibition and the introduction of the Beer–Wine Revenue Act of 1933 [13,16]. These laws and regulations created a market opportunity for 3.2% ABV beer that might not have otherwise existed; the regulatory context for beer made it appealing to craft lower alcohol by volume (ABV) versions of beers that are also made in higher ABV form. For example, you might find a 4.0% Bud Light (which is 3.2 ABW) as well as a 4.5% ABV Bud Light in the right regulatory context. However, generally speaking, these lower ABV versions disappeared rapidly once the legal landscape shifted, providing evidence that these products were propped up more by the regulatory system than consumer demand. Because these laws have largely disappeared in recent decades, the current market for lower-alcohol beers seems to be driven more by demand and encompasses a wider range of brands and flavors than earlier low-ABV products.

Despite the growing interest in low- and no-alcohol beers on the part of consumers as well as those seeking to mitigate alcohol-related health risks [2], there are still some headwinds for many producers in the United States and beyond. Globally, the definitions for, classifications of, and regulatory guidelines surrounding no- and low-alcohol products are extremely variable, despite widespread agreement among public health professionals that monitoring and potentially reducing alcohol’s impacts on society is a wise policy choice [2]. Thus, at the global scale at least, the debate is ongoing regarding how to best incentivize the production of no- and low-alcohol products [1]. In the United States, the same ambiguities and disincentives prevail. For low-alcohol beers, the US generally taxes beverage alcohol based on volume, not on ABV. That means that, in contrast to many other countries where lower ABV beers might be taxed at a lower rate [17], low ABV beers in the US are generally taxed at the same rate as higher ABV beers (as beer is generally taxed based on the volume produced) [18]. As such, while there may be savings on the cost of inputs, US tax rules reduce the incentive for the production of these products in the absence of strong consumer demand.

There are also technical challenges for small producers in terms of producing no- and low-alcohol beers, even though there are a variety of ways to do so [19]. Non-alcohol beers are defined as less than 0.5% ABV in the US tax code; beers that are truly 0.0% alcohol can label themselves as “alcohol-free” as opposed to “non-alcoholic”, and both are regulated as “cereal beverages” [20]. Methods used to achieve this level of alcohol include arrested fermentation, continuous fermentation, alternative mashing practices, the use of specific yeasts (whether *Saccharomyces* or not), or other techniques to ensure fermentation does not ferment above 0.5% alcohol [15]. Another method includes removing the alcohol [4]. Many new products on the market use vacuum distillation, wherein the alcohol is removed from the product by boiling it in a vacuum [4]. A risk with this technique is that, in the process of removing the alcohol, other phenolic compounds are also removed, impacting the beverage’s flavor. Although technology now exists that can remove alcohol at lower temperatures—allowing for the retention of more of those flavor components—the process requires specialized (and expensive) machinery that is unavailable to most small producers [4]. However, given that craft brewers are noticing this trend toward low(er) alcohol products and are, thus, looking for ways to expand into that segment, attention on how to efficiently do so, especially within these producers’ given cost constraints, is a fertile area of research in the contemporary moment [19].

In sum, low- and non-alcoholic beers are clearly a growing part of the market once more. However, at this time, the no- and low-alcohol market is being shaped by flavor and quality considerations, including a desire for the health benefits associated with certain beer ingredients (such as B vitamins, minerals, and more), especially in an alcohol-conscious customer base that largely overlaps with those who also drink full ABV products [15]. Given the growth and significance of this segment, this research examines the types, ranges, and representations of low(er) alcohol beers offered by craft breweries in the US as observed in a sample of craft breweries’ lowest ABV offerings.

## 2. Materials and Methods

To conduct this research, we curated a broad (randomized, though non-representative) sample pulled from a comprehensive list of craft breweries from across the United States, upon which we conducted a quantitatively-informed qualitative analysis. The analysis was modeled after the approach used by Myles et al. (in press) [21] and builds on research by Savelyev et al. (2019) [22] and Myles et al. (2020) [3]. This digital geography [23] entailed visiting each brewery’s website to record several pieces of voluntary data related to the lowest ABV beverage on offer. The numerical ABV, as well as those low(er) ABV beverages’ names and brew types (e.g., lager, pilsner, ale), were recorded. This quantitative data collection was complemented by the collection of website screenshots of the relevant beverages to be used for subsequent qualitative analysis.

To curate our sample, a complete list of 9100 craft breweries from the Brewers Association was generated. We omitted associate members to keep this analysis in alignment with previous studies [21]. Associate members were omitted in the original study because they could be allied trade, distributors, retail members, and non-voting breweries. A two-layer randomization technique was used for selecting 400 breweries. First, the list of 9100 was entered into Excel, and then, using the “RAND” formula, each brewery was assigned a random number in a new column. The numbers column was then sorted from smallest to largest, and the value was locked in the cell by copying and pasting special, as value. Next, the top 1200 were labeled as A, B, or C to sort into groups of 400. Finally, all the Bs were selected for analysis. 

Thusly, out of the full list of 9100 breweries, a randomized list of 400 was pulled for sampling, based on the sampling methodology used in a previous study [21], to garner a 95% confidence level. While we pulled what we thought would be a representative sample of breweries, only 296 of the 400 proved worthy of inclusion. There were several reasons that a brewery could be excluded from analysis, including the brewery being out of business (14), not producing beer (10), having no website or a non-functioning website (18), using a social media site rather than a traditional website (3), or, importantly, including no information about the beers on offer or their ABVs (59). 

Even though the sample used here is not truly representative, which is due not to a shortage of breweries surveyed (ordinarily, a sample of 369 would suffice to represent a population of 9100; we surveyed 400) but, rather, reflects a gap in the data provided by the breweries themselves (namely, the ABV of their beers or, in some cases, any details at all about their beer), it is robust, and our findings remain valid as this is intended as an exploratory study. As such, the research design could be replicated using a larger sample to produce representative results.

Data collection involved visiting the internet home of the 400 randomly selected breweries. As noted, a number of breweries (108, to be exact) were removed from analysis due to having a non-suitable web presence or no longer being in operation. For the valid sites, we identified and recorded the lowest ABV offering, the beer type, and the beer name. This research was conducted over a period of ten days (14–23 June 2022). The ABV and type were recorded in Excel, while the names were logged in a Word document. Beer type is determined by the Brewer’s Association 2022 Beer Style Guidelines [24]. For example, if an offering is listed as a Kolsch, it is recorded as an ale because Kolsch beers fall under the ale category of the style guidelines. 

The style guidelines include three main beer types: ale, lager, and hybrid/original. However, some of the low(er)-ABV records fell outside of the style guide (e.g., cider, seltzer) and were instead placed into a separate fourth category. The types were assigned numbers (1 for ale, 2 for lager, 3 for hybrids and originals, and 4 for “other”) and recorded in the Excel document. Within Excel, averages were calculated, and types were sorted. 

**Figure 1:**
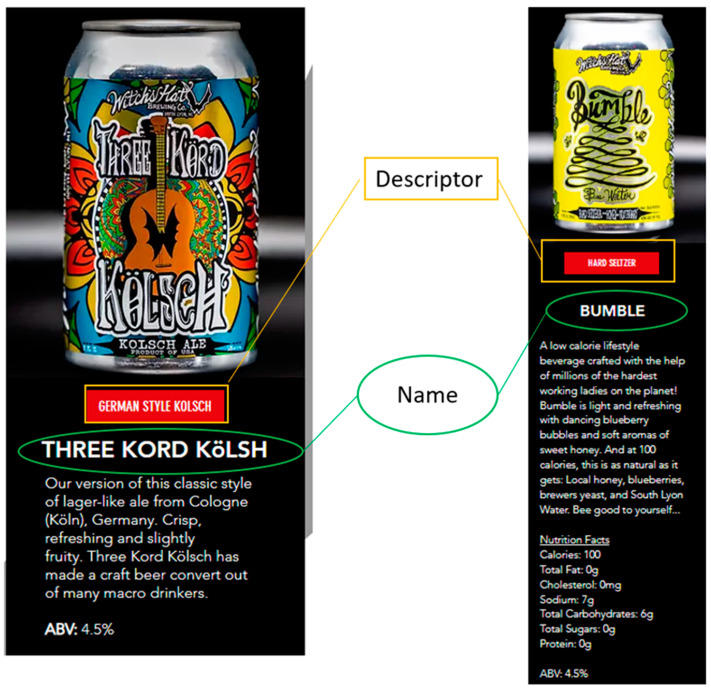
Examples of names and descriptors used for branding craft brewery offerings. (Screenshots collected by the authors from [https://www.witchshatbrewing.com/; accessed on 30 July 2022]).

The names of the lowest-ABV beverage identified on each brewery’s website and the style descriptors used to describe them (Figure 1) were recorded for analysis. We then used Voyant Tools (https://voyant-tools.org; accessed on 3 October 2022), a web-based reading and analysis environment for digital text, to create word cloud visualizations for the offering names and descriptors found on the breweries’ websites. Given that these two different sets of data each created a different “corpus” (set of words) for analysis, the resulting visualizations also vary, as presented in the Results section.

## 3. Results

### 3.1. How Low Can You Go? (Low) ABV Breakdown

**Figure 2:**
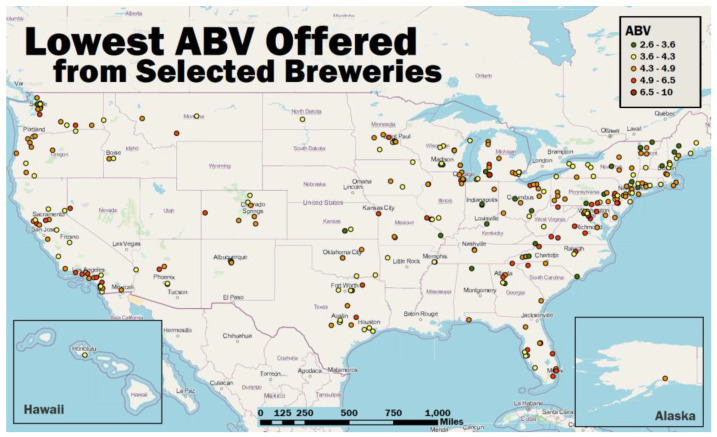
Map of included breweries’ locations and lowest ABV on offer. (Map by AUTHOR and Heather Swienton; 17 November 2022).

Across the 400 craft breweries examined, 296 yielded positive results (positive result = website provided ABV values). Figure 2 depicts the locations and lowest ABV on offer for each of the breweries included in this study. The average lowest ABV offering was 4.42% (Table 1). Values ranged from 0–10% ABV. This range includes two outliers: a 0% “hop water” (a non-beer using hops) and a 10% mead. Once these two are removed, the range tightens to 2.6–6.5% ABV. Forty-six results were <4% ABV, with the average being 3.47% ABV. The remaining 250 offerings of >3.9% ABV averaged 4.6% ABV. However, if the outlying 10% ABV offering is removed, the average drops to 4.58% ABV. We did not conduct a significant difference check as this is not a representative sample of breweries in the US.

### 3.2. What’s Your Type? Beer Types and Styles

**Figure 3:**
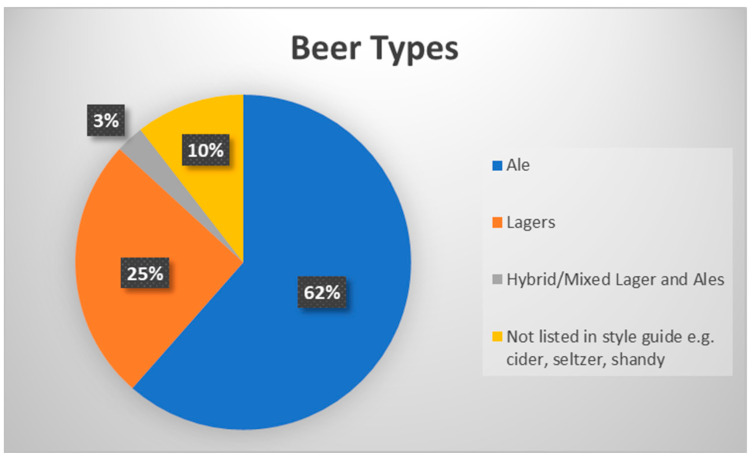
Beer type by percentage. (Data from the authors; pie chart constructed in Excel).

Our survey of brewery websites revealed that breweries use a variety of terms to describe their brews, but mainly those terms conform with the mainstream styles outlined by the Brewers Association [24]. The types offered by the websites surveyed included “Ales” (182), “Lagers” (75), “Hybrid/Mixed Lager and Ales” (8), and several outliers styles (31) that were not listed in style guidelines (e.g., cider, seltzer, shandies) (Figure 3).

**Figure 4:**
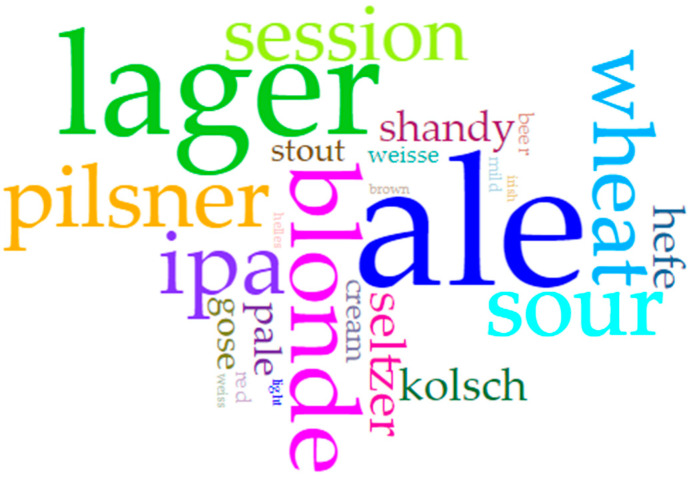
Word cloud (larger text indicates higher frequency of use) visualizing the frequency of words in breweries’ descriptions of their low(er) alcohol offerings (Data from the authors; visualization via Voyant: https://voyant-tools.org/?corpus=aef5201d283dc3b70ed5289ab57d42e2; accessed on 3 October 2022).

We created a word frequency analysis (word cloud) to depict the variety of terms used to describe the styles of beers captured in our survey (Figure 4). In it, “ale” (76), “lager” (51), “blonde” (26), “IPA” (24), and “pilsner” (23) stand out. Some of these styles are known to be traditionally lower in alcohol [25], so this finding mainly comports with expectations. However, the prominence of the term “ale” is noteworthy given that “light” lagers have long driven lower ABV trends in the US [9,13], though this may be due more to regulatory requirements by the state rather than true style concerns [3]. Moreover, lager production requires (more) expensive equipment and a greater commitment of time for production, amounting to a set of true and opportunity costs that many smaller craft breweries cannot bear. As such, ales are more common among these types of producers. That being said, the relative infrequency of the term “session” (though at 18 mentions, the term is not far behind the leaders) is also surprising, given its rise in popularity in recent years as consumers have tapped into the lower-ABV segment [26,27]. Nevertheless, the styles observed in this survey are in line with the (re)emergence of a subset of beers known as “soft” or “small” ales [3,5]. Future research could examine whether the prominence of ales versus lagers varies along any particular dimensions within the population of craft breweries (e.g., geographic factors, latitude or climate, cultural context, etc.).

### 3.3. Who’s That? Beer Names

**Figure 5:**
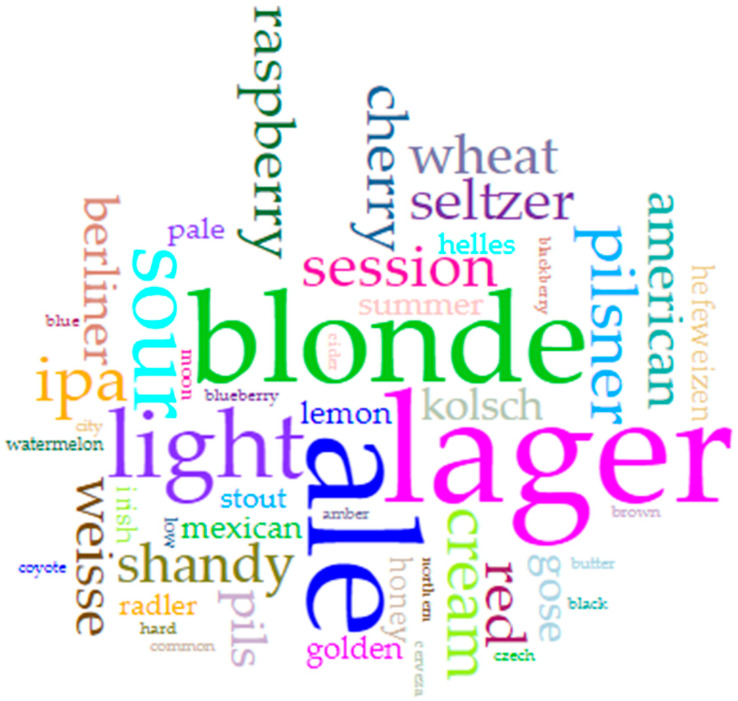
Word cloud (larger text indicates higher frequency of use) visualizing the frequency of word choice in the names of the lowest ABV offerings from the breweries surveyed. (Data from the authors; visualization via Voyant: https://voyant-tools.org/?corpus=51da88b672374ebe39516cd3f27ad752); accessed on 3 October 2022.

The results related to beer naming conventions show that, of 798 words used to describe the lowest ABV beverage on offer, “ale” (29), “lager” (29), “blonde” (19), “light” (14), and “sour” (11) appear most prominently (Figure 5), mirroring the findings related to beer styles. Notable also is the visibility of fruit words (e.g., raspberry, cherry, lemon). Future research could assess the motivations or drivers of this component of the low(er) alcohol beer naming conventions. An additional study might consider whether and how lower alcohol beers lean on fruit flavors to improve taste or enhance otherwise less flavorful or robust brews, if a fruity component to a beer is expected to draw the attention of particular consumer groups, or if fruit-driven flavors are more common in some seasons (i.e., summer versus winter) or styles (i.e., gose).

**Figure 6:**
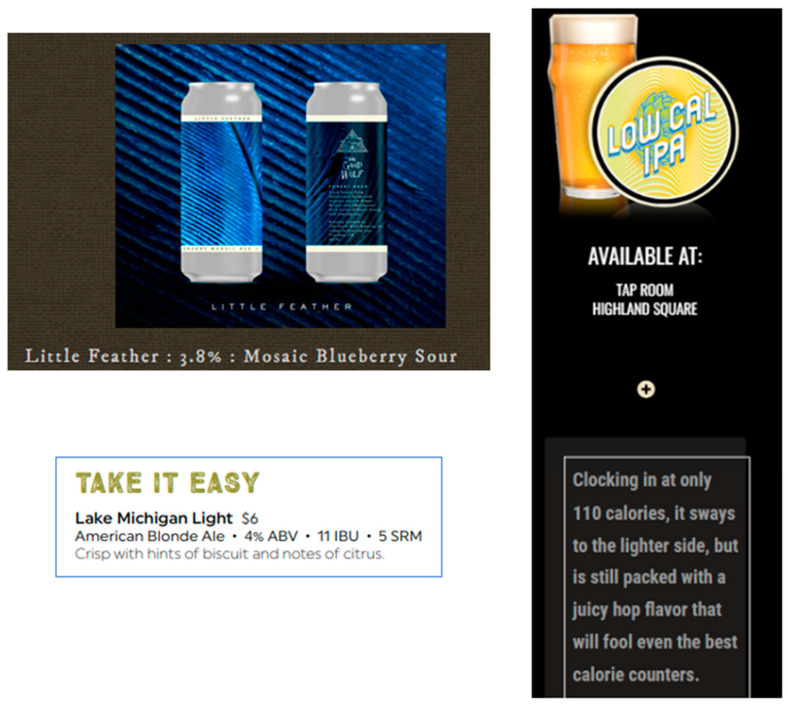
Examples of how low ABV offerings are represented. (Screenshots collected by the authors from http://www.thegoodwolfbrewing.com/, https://www.schlafly.com/, and https://roundbarn.com/pubhouse; accessed on 30 July 2022).

Our qualitative review of brewery websites provided insights regarding the iconography and general representation of low(er) alcohol offerings. Broadly speaking, as seen in Figure 6, words and phrases like “low calorie,” “[on] the lighter side, but…still packed with flavor,” “little feather,” and “take it easy” are used to describe these low(er) alcohol beers. Imagery evoking lightweight and diminutive objects and concepts are clearly paired with invocations of flavor and character—revealing a clear desire to move past the pale, bland stereotype of mass market light(er) beers as well as to appeal to those seeking a “lighter” or “healthier” beer option.

**Figure 7:**
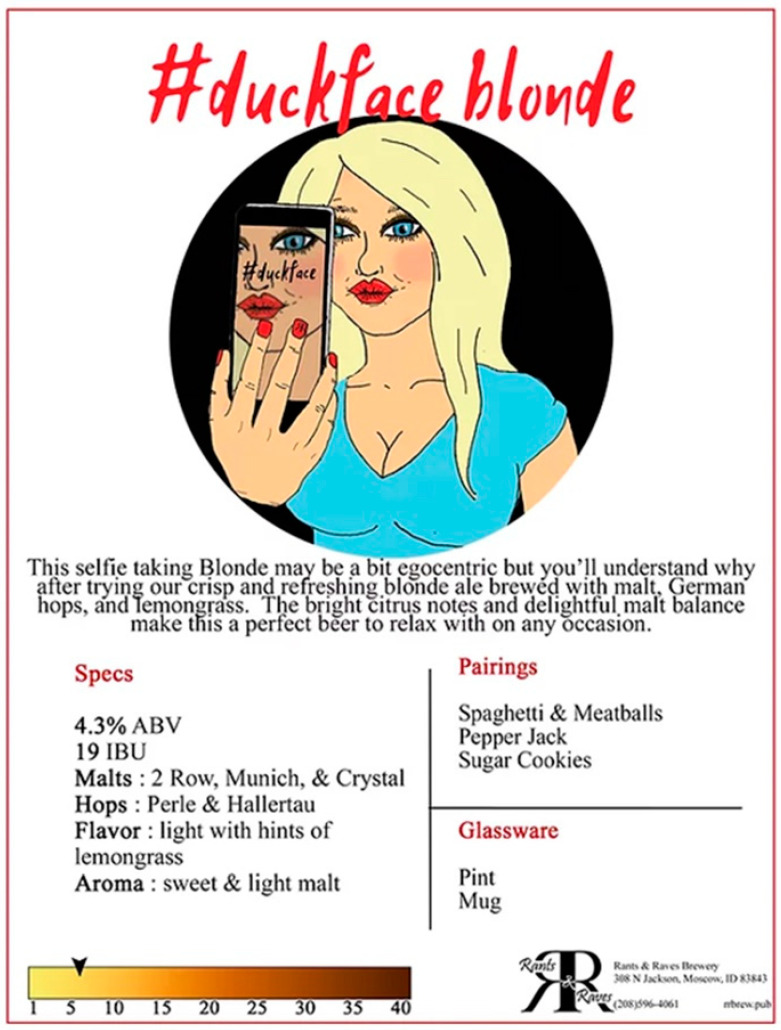
A particularly striking example of how a low ABV offering is represented by a craft brewery. (Screenshot by the authors from https://www.rantsravesbrewery.com; accessed on 30 July 2022).

A striking example of how these different concepts are put into play is offered by the “#duckface blonde” by Rants and Raves Brewing (https://www.rantsravesbrewery.com; accessed on 30 July 2022). The full description (Figure 7) reads: “This selfie taking Blonde may be a bit egocentric but you’ll understand why after trying our crisp and refreshing blonde ale brewed with malt, German hops, and lemongrass. The bright citrus notes and delightful malt balance make this a perfect beer to relax with on any occasion.” This beer description is packed with both production and tasting notes, as well as a fair bit of social commentary. Interestingly, the description for this beer changed sometime between July 2022 and September 2022 during data collection and analysis. The more recent descriptor reads: “Crisp and refreshing blonde ale spiced with lemongrass. The bright citrus notes and delightful malt balance make this the perfect beer to relax with on any day.” The updated description still alludes to when and with whom you might want to enjoy a beer like this, but a bit less aggressively. Instead, the message offers a slightly different take, suggesting that this beer is one to “relax” with “on any day,” inviting the attention of health-conscious drinkers and/or those who are potentially trying to moderate their overall intake.

## 4. Discussion

While many brewery websites provide a list of beers produced, including their styles and ABVs, many breweries do not. We selected a sample, based on previous research, that we expected to be representative of craft breweries in the US; however, due to the number of websites that did not offer the data needed for this study, our sample turned out to be non-representative. The analysis was further complicated by the fact that some breweries offer more than one brew at the same ABV, which makes an approach based on selecting the “lowest ABV” beer potentially problematic. Furthermore, as this data was collected only during the summer season, we cannot say whether or how seasonality impacts brewer’s choices related to low(er) ABV offerings. Nevertheless, as this study is exploratory—with no other research existing on this topic to date—our findings are still worthwhile in terms of discovering broad trends in this segment of the brewing landscape.

Our preliminary analysis revealed that, at least among the breweries sampled, these businesses market their products based on taste (i.e., blueberry sour), health indicators (i.e., “low cal” IPA), and demographic profiling (i.e., Duckface Blonde). Further research (on an expanded sample to ensure representativeness) could delve more fully into the qualitative components of this data. From the analysis conducted here, however, we can draw some conclusions. In terms of what is being offered, ales were represented more than other styles, accounting for 62% of the low(er) ABV beers identified. Other prominent styles were lagers and blondes. Although lagers are often lower in ABV in general, fewer craft breweries produce them due to the technology and time required, so the relative abundance of ales is not surprising given that this study is focused on smaller-scale craft producers.

Of the 400 websites analyzed for this project, 296 provided ABV values. The average ABV of the lowest ABV offerings on each website was 4.42%, with a range of 2.6–6.5% ABV (once you exclude two outliers, a 0% ABV beverage and a 10% one). A total of 46, or 15.5%, of the 296 breweries surveyed offered a beer with an ABV lower than 4%, with an average of 3.47% ABV. The remaining 250 low(er) ABV offerings averaged 4.6% ABV. This analysis suggests that many craft breweries in the US do offer something that could be considered a low(er) alcohol product, given that, at least within this sample, the average ABV of the lowest-ABV brews on offer hovered around 4.5%, which is, notably, the same as it was in 1934, just after Prohibition [13].

While there is no official definition of a low-alcohol beer in the United States, 3.2 ABW (4% ABV) acts as a functional guide. While just 15.5% of the breweries surveyed in this study offered a beer with an ABV of less than 4%, an additional 201 (of 296), or 67.9%, offered a beer with an ABV of less than 5%. If you include those beers that are at or below 5% ABV—a level of alcohol that could reasonably be considered low enough in alcohol to be reliable for a several-beverage drinking “session”) [24,25,27]—the number of breweries in this sample producing “sessionable” beers (a popular format for the consumption of lower-alcohol beers) is a whopping 89.5% (265 out of 296).

In conclusion, the low(er) alcohol (craft) beer segment appears to be alive and well, which is good news to anyone seeking to promote its consumption for health and wellness or any other reason. Future research on this topic will include a repeat of this study using a representative sample. In addition, a consumer-based study, conducted in the United States or globally, to investigate what styles, attributes, or marketing related to low(er) alcohol beer are the most appealing and why would also be valuable.

## Figures and Tables

**Table 1:** Summative ABV data; the range provided excludes the two outliers (two beverages with 0% and 10% ABV) in order to provide a more accurate sense of the real landscape of these low(er) ABV offerings. (Data from the authors).

Total Breweries Examined	400
**Positive results**	296
**Average lowest ABV**	4.42%
**Range**	2.6–6.5%

## Data Availability

This study was conducted using publicly available information on the web.
